# Association of oil spill cleanup-related hydrocarbon exposure with incident hypertension up to 11 years after exposure in the Gulf Long-term Follow-up Study

**DOI:** 10.1186/s12940-025-01253-9

**Published:** 2025-12-30

**Authors:** Opal P. Patel, Jessie K. Edwards, Anna M. Kucharska-Newton, Eric A. Whitsel, Kate E. Christenbury, W. Braxton Jackson II, Kaitlyn G. Lawrence, Patricia A. Stewart, Mark R. Stenzel, Lawrence S. Engel, Dale P. Sandler

**Affiliations:** 1 Department of Epidemiology, UNC Gillings School of Public Health, Chapel Hill, NC USA; 2 Epidemiology Branch, National Institute of Environmental Health Sciences, National Institutes of Health, Research Triangle Park, NC 27709 USA; 3 Department of Medicine, UNC School of Medicine, Chapel Hill, NC USA; 4 Social & Scientific Systems, Inc., a DLH Holdings Company, Durham, NC USA; 5 Stewart Exposure Assessments, LLC, Arlington, VA 22207 USA; 6 Exposure Assessment Applications, LLC, Arlington, VA 22207 USA

**Keywords:** Volatile hydrocarbons, Hypertension, Oil spill, Environmental epidemiology

## Abstract

**Background:**

While several studies have found positive associations between exposure to oil spill cleanup-related chemicals and hypertension, no study has examined these associations longitudinally.

**Objective:**

This study examined associations of oil spill-related benzene, toluene, ethylbenzene, xylene, and n-hexane (BTEX-H) exposures, individually and as both the aggregate sum (total) of BTEX-H and the BTEX-H *mixture* with incident hypertension among Gulf Long-term Follow-up (GuLF) Study participants.

**Methods:**

Participants were 18,619 *Deepwater Horizon (DWH)* oil spill cleanup and response workers who enrolled in the GuLF Study (2011–2013). Cumulative exposures to each BTEX-H chemical were estimated with a job-exposure matrix linking detailed self-reported *DWH* participant work histories to exposure group estimates developed from air monitoring data. We defined incident hypertension as the first self-reported physician diagnosis of hypertension or high blood pressure after each worker’s last date of cleanup work, as reported at enrollment or a follow-up interview (2013–2016 or 2017–2021). We used Cox proportional hazards regression to estimate hazard ratios (HR) and 95% confidence intervals (CI). We used quantile g-computation to estimate the joint effect of the BTEX-H *mixture*.

**Results:**

Approximately 20% (*n* = 3,779) of workers reported an incident hypertension diagnosis. Exposures to the individual BTEX-H chemicals were highly correlated (*r* = 0.87–0.95). The HRs comparing the highest to lowest quartiles of individual BTEX-H and total BTEX-H exposures ranged from 1.27 to 1.35. We found evidence of exposure-response trends across increasing quartiles of exposure. Each one quartile increase in the BTEX-H *mixture* was positively associated with incident hypertension (HR: 1.10, 95% CI: 1.07, 1.14).

**Discussion:**

Oil spill cleanup work-related BTEX-H exposures were associated with the risk of incident hypertension, extending prior findings of cross-sectional associations. Since BTEX-H exposures are common in occupational and population settings, these findings may have broader public health implications.

**Supplementary Information:**

The online version contains supplementary material available at 10.1186/s12940-025-01253-9.

## Introduction

The *Deepwater Horizon (DWH)* disaster on April 20, 2010, was the largest marine oil spill in United States (US) history [1]. An extensive oil spill response and cleanup (OSRC) effort involved workers from the Gulf states and across the US[1]. The OSRC tasks exposed workers to crude oil containing total hydrocarbons (THC) including benzene, toluene, ethylbenzene, xylene, and *n*-hexane (BTEX-H)[2–10].

THC and BTEX-H exposures have been associated with cardiovascular disease (CVD) risk but few epidemiological studies have examined specific associations of BTEX-H chemicals with blood pressure (BP) and hypertension [11–20]. Earlier analyses of GuLF Study participants found that THC and BTEX-H exposures were positively associated with the prevalence of hypertension and systolic and diastolic BP at the time of a home visit up to 3 years after exposure, but did not consider longer-term risk [13, 21]. Other studies reported positive associations of total volatile organic compound (VOC) exposure (of which THC is a component) with BP and hypertension but did not examine specific chemical constituents of VOC [22–25]. These studies had a number of important limitations, including cross-sectional designs or small sample sizes, which limited causal inference, statistical power, and ability to account for potential confounders [22–25]. 

While existing literature provides evidence that BTEX-H exposures are associated with hypertension cross-sectionally or over a short time period, no study to date has examined these associations longitudinally. The current study aimed to assess associations of BTEX-H chemicals, individually, combined as an aggregate sum (total BTEX-H), and as a mixture, with self-reported hypertension up to 11 years after exposure among *DWH* OSRC workers.

## Methods

### Study population

The Gulf Long-term Follow-up (GuLF) Study enrolled 32,608 adults aged 21 years and older who engaged in work related to the response and cleanup of the *Deepwater Horizon (DWH)* disaster in 2010 (workers) or who received worker safety training but did not work (non-workers) [5]. Study participants were enrolled via telephone interviews between March 2011 and March 2013 and provided information on demographics, lifestyle, health, and a detailed work history of *DWH* oil spill response and cleanup (OSRC) activities. After enrollment, all English- and Spanish-speaking participants were invited to participate in two follow-up telephone interviews (May 2013-April 2016 and November 2017-July 2021) to ascertain any changes in health status since the previous interview. Of the 32,608 who enrolled into the study, 21,248 completed the first follow-up interview and 14,178 completed the second follow-up interview, with 12,858 participants completing both.

For the current study, we excluded 999 Vietnamese-only speaking participants who completed an abbreviated enrollment interview in which they were not asked about hypertension diagnosis. We restricted our analysis to the 24,375 workers at enrollment, since the study did not estimate BTEX-H exposures for non-workers. Start of follow-up was defined as each worker’s last date of cleanup work. We excluded 5,203 workers who reported a first hypertension diagnosis prior to the start of follow-up or whose first diagnosis relative to start of follow-up could not be determined. We further excluded workers who did not report an end date of cleanup work (*n* = 156) or were missing key study covariates (*n* = 379), for a final analytical sample of 18,619 workers.

#### Human subjects

All participants provided informed consent. This study was reviewed and approved by the Institutional Review Board of the National Institutes of Health.

### Exposure assessment

The process for estimating inhalation BTEX-H exposures has been described in detail elsewhere [3, 4, 7, 26–28]. *DWH* cleanup-related exposure to each individual BTEX-H chemical and to total BTEX-H were estimated via a job-exposure matrix that linked air measurement data to the self-reported OSRC work history of each participant. Briefly, air measurement data came from over 28,000 personal exposure samples collected on OSRC workers via organic vapor passive dosimeters worn during OSRC work between April 2010 and June 2011. Laboratory assays of these samples resulted in over 143,000 measurements of total hydrocarbons (THC) and BTEX-H. Measurement results reported as less than the limit of detection (LOD) were converted to detectable values if the reported value was higher than the actual analytic LOD [29]. Additionally, direct-reading volatile organic compound (VOC) area measurements were collected from 38 large vessels involved in the cleanup. Study industrial hygienists converted these measurements into the equivalent full-shift THC and BTEX-H exposure estimates [26, 30] to supplement days when there were few or no personal measurements on those particular vessels[26, 30].

To estimate inhalation exposures, over 3,000 exposure groups were created by the industrial hygienists based on three determinants: self-reported job/activity/task (hereafter called job; e.g., skimming water to collect surface oil, burning surface oil); location of work, based on increasing distance from the well head (hot zone/source, offshore, near-shore, and land) and four Gulf coastal states (Louisiana, Mississippi, Alabama, Florida); and time period (seven time periods over 14 months that captured changes in OSRC events and oil weathering) [6]. Each exposure group comprised monitored clean-up workers with a unique combination of these three determinants and who were expected to have similar exposure distributions. Due to the large number of measurements below the analytical method’s limit of detection for some exposure groups, a left-censored Bayesian framework incorporating THC measurements (which had the fewest measurements below the limit of detection) was used to estimate individual BTEX-H and total BTEX-H[10, 27]. The arithmetic mean of the air measurements was estimated for each corresponding exposure group, in parts per billion (ppb). Total BTEX-H (the sum of the five individual BTEX-H ppb values) was calculated for each exposure group and represents an aggregate VOC exposure specifically due to BTEX-H chemicals. Each study participant’s self-reported *DWH* OSRC work history was linked to the appropriate exposure groups to assign participant BTEX-H exposure estimates.

Because participants often reported performing multiple jobs over the same day, workers were assigned a daily average (the average of the exposure estimates across all jobs on each day) and a daily maximum (the value corresponding to the highest exposed job on a given day) exposure for each day worked, in ppb-days. The quality of information on the hours spent performing each of these jobs did not allow for calculating weighted daily exposure estimates. For the current report, we used cumulative daily maximum exposure to individual BTEX-H chemicals and total BTEX-H as the primary exposure metric. Supplementary analyses also considered the cumulative daily average exposures.

### Outcome assessment

We classified hypertension cases based on the first self-reported physician diagnosis of hypertension after the last day of each participant’s OSRC work. Participants were asked if they had ever received a physician diagnosis of hypertension at enrollment (March 2011-March 2013), the first follow-up (May 2013-April 2016), and the second follow-up (November 2017-July 2021) interviews. Those who responded “yes” were asked to provide the month and year of diagnosis or their age at diagnosis. For those who provided age at diagnosis, the date of diagnosis was estimated as the midpoint of that age year. For participants who provided discrepant hypertension diagnosis dates during multiple interviews (*n* = 1,038), we used the first reported date of diagnosis. For diagnoses reported in the enrollment interview as after the end of clean-up but with date missing or uncertain ages at diagnosis (*n* = 6), we assigned the midpoint date between start of follow-up and enrollment. We assigned the midpoint date between visits (*n* = 279) for participants who responded “no” to having a hypertension diagnosis at an earlier interview but reported a hypertension diagnosis with no date or age at diagnosis at a subsequent interview. We assigned midpoints to 92 participants between enrollment and first follow-up, 33 participants between enrollment and second follow-up, and 154 participants between first and second follow-up.

We measured time at risk in months from each participant’s last date of cleanup work to the first date of self-reported hypertension diagnosis, death from other causes, or end of active follow-up (July 31, 2021). For individuals who were lost to follow-up, we used the day after the date of the last completed interview to calculate the risk period. Death was ascertained via linkage with the National Death Index.

### Covariate assessment

Using a directed acyclic graph, we identified potential confounders of the relationship between oil spill cleanup-related BTEX-H exposures and incident hypertension to include in a minimally sufficient adjustment set [31]. Variables adjusted for included age (continuous, with quadratic splines at ages 50 and 60 years), sex (male, female), self-reported race (White, Black, Other) and Hispanic ethnicity (Hispanic, non-Hispanic), highest educational attainment (less than high school, high school diploma/General Education Development, some college/2-year degree, 4-year college graduate or more), BMI (< 18.5, 18.5–24.9, 25.0–29.9.0.9, ≥ 30.0 kg/m^2^), and residential proximity to the oil spill (living in a county or parish directly adjacent to the Gulf of Mexico, a county/parish adjacent to those coastal counties, a Gulf county or non-Gulf state further from the spill). All covariates were ascertained at enrollment; body mass index (BMI) was calculated using self-reported weight (kilograms) divided by height (meters) squared (kg/m^2^). Self-reported weight has previously been shown to be an accurate proxy of measured weight values [32]. We evaluated self-identified race and Hispanic ethnicity as potential confounders since factors driven by lived experience (such as residential location and employment) may have separately influenced exposure opportunity to oil spill cleanup-related compounds or other potential influential exposures and to health care access for a physician diagnosis of hypertension or high blood pressure [33–35]. We visually assessed smooth Loess graphs to determine the functional form utilized for continuous variables in multivariable regression. Due to small sample sizes, we combined race and ethnicity into one variable and grouped those who self-identified as Asian (*n* = 172), non-Hispanic Other (*n* = 955), or Hispanic (*n* = 1,299) into an “other” race and ethnicity category.

We assessed neighborhood disadvantage, as measured by the 2013 Area Deprivation Index (ADI), as a potential effect measure modifier [36, 37]. The 2013 ADI is a quantitative measure of neighborhood disadvantage (linked to participant enrollment addresses at the census block group level) using 17 poverty, education, housing, and employment indicators from the American Community Survey [36, 37]. These indicators are weighted using factor score coefficients to assign a percentile ranking of 1 to 100 for lowest to highest disadvantage, respectively.

### Statistical analysis

We report descriptive statistics as means, counts, and percentages. Individual BTEX-H and total BTEX-H were categorized into quartiles of cumulative maximum exposure to capture non-linear exposure-response relationships (Supplementary Table 1). We modeled exposure quartiles as disjoint indicator variables, with subjects in the lowest exposure quartile as the referent group. We used Cox proportional hazards regression to estimate hazard ratios (HR) and 95% confidence intervals (CI) for the associations of each individual BTEX-H chemical and total BTEX-H with incident hypertension up to 11 years after each worker’s last date of cleanup work. We also used quantile g-computation to estimate hazard ratios corresponding to the joint effect of the BTEX-H *mixture *using R package version 2.9.0 “qgcomp”[38]. We used months since exposure (each worker’s last date of cleanup work) as the time scale. Tests of exposure-response trends were conducted by modeling exposure quartiles continuously. All analyses were conducted with R Studio version 4.3.1.

In the main analysis, we adjusted for age, sex, self-reported race and Hispanic ethnicity, highest educational attainment, BMI, and residential proximity to the oil spill. We investigated potential effect measure modification by age (< 40, ≥ 40 years), sex (male, female), race and ethnicity (non-Hispanic White, non-Hispanic Black, Hispanic), obesity (non-obese (BMI < 30 kg/m^2^), obese (BMI ≥ 30 kg/m^2^)), and neighborhood disadvantage (low vs. high neighborhood disadvantage) using stratified models to evaluate effects within subgroups. Age was dichotomized at the cohort median to assess potential variation in effect among younger versus older age participants. The “non-Hispanic Other” race and ethnicity category was not included because of small numbers and the diversity of groups within this sample. We dichotomized neighborhood disadvantage based on the US percentile to examine differences in associations in stratified analyses of participants with low (1-75th) versus high (76-100th) neighborhood disadvantage.

### Sensitivity analyses

We separately assessed associations of BTEX-H exposures as the cumulative daily average (the daily average exposure estimates across all jobs a worker performed in a given day, summed across all days worked). In separate sensitivity analyses, we excluded 1,379 individuals who provided conflicting hypertension diagnosis dates or did not provide a diagnosis date (and were, therefore, assigned a midpoint date). We applied discrete time assumptions in which we assigned the date of the interview at which the first hypertension diagnosis was reported (instead of the self-reported or imputed diagnosis date) given the potential for inaccurate recall and observed discrepancies in self-reported hypertension diagnosis dates. To assess the potential impacts of left truncation that would have occurred if workers developed hypertension after their last day of OSRC work and were subsequently unable to enroll into the GuLF Study, we repeated analyses with a risk period starting at each participant’s enrollment date (instead of the participant’s last date of cleanup work). This resulted in the exclusion of 1,590 individuals diagnosed with hypertension between the end of cleanup and their enrollment, for an overall sample of 12,444. We conducted an analysis using inverse probability censoring weights (IPCW) to evaluate the potential bias introduced by censoring those lost to follow-up in our main analyses. We also repeated analyses excluding participants who worked at the source or hot zone who were in oil spill professions and likely had prior exposures, greater access to protective gear, and better access to medical care, as well as those who worked on in situ burning of the oil, whose exposure to particulate matter with aerodynamic diameter ≤ 2.5 μm (PM_2.5_) was ≥ 10.4 ug/m^3^ from controlled burning activities during oil spill cleanup (*n* = 1,697) to account for potential confounding from this exposure. In separate sensitivity analyses, we restricted to never smokers (*n* = 9,120) to account for potential confounding by smoking exposures among former and current smokers.

## Results

Selected characteristics of participants in our analytic sample are reported in Table 1. The mean age of participants was 40 (SD ± 12.1) years, and most were male (82%) and Non-Hispanic White (65%) individuals. Approximately half of participants had attained high school education or less (45%) or were never smokers (50%). Overall, no substantive differences were observed in most characteristics between the full analytical sample and those who completed the first and/or second follow-up interview (in addition to the enrollment interview) (Supplementary Table 2). The only differences we observed were among the second follow-up interview participants, who were more likely to be Non-Hispanic White and to have higher educational attainment compared to the full analytical sample. We found that individual BTEX-H exposures for cumulative maximum exposures were strongly correlated (*r* = 0.87–0.95) (Supplementary Table 3).

**Table 1:** Characteristics of analytic sample of GuLF study participants, 2010–2021

Characteristic	Overall	Hypertensive^a^	Non-hypertensive
	*n* = 18,*619*	*n* = 3,*779 (20.3%)*	*n* = 14,*840 (79.7%)*
	***Mean ± SD***	***Mean ± SD***	***Mean ± SD***
Age (continuous)	40.0 ± 12.1	44.7 ***±*** 11.9	38.8 ***±*** 11.8
	***N (%)***	***N (%)***	***N (%)***
Age			
<40 years	9,572 (51.4)	1,291 (34.2)	8,281 (55.8)
≥40 years	9,047 (48.6)	2,488 (65.8)	6,559 (44.2)
Sex			
Male	15,211 (81.7)	3,161 (83.7)	12,050 (81.2)
Female	3,408 (18.3)	618 (16.4)	2,790 (18.8)
Education			
Less than high school	2,786 (15.0)	698 (18.5)	2,088 (14.1)
High school diploma/GED	5,484 (29.5)	1,185 (31.4)	4,299 (29.0)
Some college/2 year degree	5,593 (30.0)	1,148 (30.4)	4,445 (30.0)
4 year college graduate or more	4,756 (25.5)	748 (19.8)	4,008 (27.0)
Race and ethnicity^b^			
Non-Hispanic White	12,013 (64.5)	2,225 (58.9)	9,788 (66.0)
Non-Hispanic Black	4,149 (22.3)	1,042 (27.6)	3,107 (20.9)
Non-Hispanic Other	1,138 (6.1)	229 (6.1)	909 (6.1)
Hispanic White	417 (2.2)	79 (2.1)	338 (2.3)
Hispanic Black	72 (0.4)	17 (0.4)	55 (0.4)
Hispanic Other	830 (4.5)	187 (4.9)	643 (4.3)
Smoking status			
Never smoker	9,120 (49.6)	1,660 (44.4)	7,460 (50.9)
Former smoker	3,626 (19.7)	861 (23.0)	2,765 (18.9)
Light current smoker	3,807 (20.7)	817 (21.9)	2,990 (20.4)
Heavy current smoker	1,837 (10.0)	401 (10.7)	1,436 (9.8)
BMI (kg/m^2^)^c^			
<18.5	146 (0.8)	14 (0.4)	132 (0.9)
18.5–24.9	5,510 (29.6)	693 (18.3)	4,817 (32.5)
25–29.9.9	7,823 (42.0)	1,538 (40.7)	6,285 (42.4)
≥30	5,140 (27.6)	1,534 (40.6)	3,606 (24.3)
Neighborhood Disadvantage^d^			
Low	13,747 (73.8)	2,601 (68.8)	11,146 (75.1)
High	4,872 (27.2)	1,178 (32.1)	3,694 (25.9)
Risk period^e^			
0–1.9.9 years	6,333 (34.0)	2,010 (53.2)	4,323 (29.1)
2–3.9.9 years	4,123 (22.1)	792 (21.0)	3,331 (22.4)
4–5.9.9 years	1,444 (7.8)	482 (12.8)	962 (6.5)
≥6 years	6,719 (36.1)	495 (13.1)	6,224 (41.9)

^a^Self-reported doctor diagnosis of hypertension or high blood pressure at enrollment (March 2011-March 2013), the first follow-up (May 2013-April 2016), or the second follow-up (November 2017-July 2021) interview

^b^Due to small sample sizes, we combined those who identified as Asian, other, or multiracial into the “other” race category

^c^BMI = body mass index; calculated as weight in kilograms divided by height in meters squared (kg/m^2^) as reported at enrollment

^d^Neighborhood disadvantage defined using the 2013 Area Deprivation Index (ADI) for low (US percentile 1-75th) and high (US percentile 76-100th) neighborhood disadvantage

^e^The risk period (years) was the time from each participant’s end of cleanup date to the earliest of first self-reported hypertension diagnosis date, death from other causes, or end of cohort follow-up.

During follow-up of up to 11 years (median 3.3 years), 3,779 (20.3%) out of 18,619 workers self-reported an incident hypertension diagnosis, with 1,251 (6.7%), 1,333 (7.2%) and 1,191 (6.4%) reported at enrollment, the first follow-up, and the second follow-up interviews, respectively. The mean time to hypertension diagnosis was 2.7 years. Hypertensive individuals were more likely to be older, Non-Hispanic Black race and ethnicity, and obese than non-hypertensive participants.

**Fig. 1:**
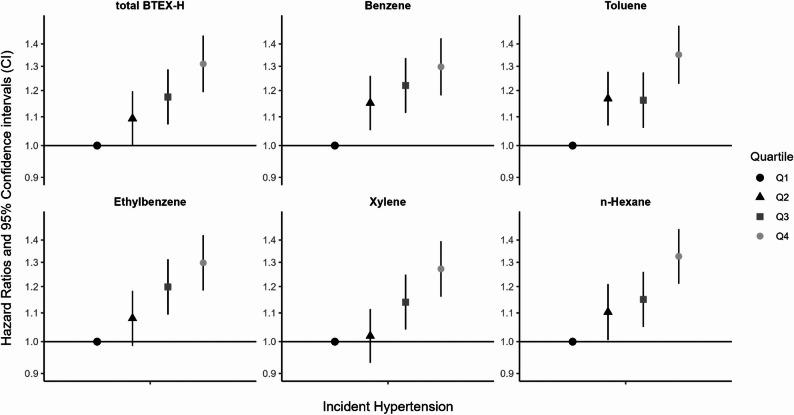
Associations between *cumulative maximum* exposure to BTEX-H chemicals and hazard of hypertension among *DWH* disaster oil spill response and cleanup workers (*n* = 18,619).^a, b, c, d^ ^a^Cox proportional hazards regression models adjusted for age (with quadratic splines at ages 50 and 60 years), sex, self-reported race, Hispanic ethnicity, highest educational attainment, body mass index, and residential proximity to the oil spill ^b^Cumulative exposure to each BTEX-H chemical measured in ppb-days, calculated as the sum of the daily maximum exposure estimates. Total BTEX-H calculated as a sum of the individual BTEX-H ppb values ^c^Incident hypertension defined as the first self-reported doctor diagnosis of hypertension or high blood pressure at enrollment (March 2011-March 2013), the first follow-up (May 2013-April 2016), or the second follow-up (November 2017-July 2021) interview ^d^Tests of exposure-response trends were assessed by modeling exposure quartiles continuously. P-trends < 0.01 were observed for associations of total BTEX-H and of each individual BTEX-H chemical with incident hypertension

Increasing levels of BTEX-H and total BTEX-H cumulative maximum exposures were associated with increased risk of hypertension, with evidence of statistically significant exposure-response trends (Fig. 1; Supplementary Table 4). For all BTEX-H exposures, we saw the strongest associations in the top quartiles (range of HRs: 1.27–1.35). When we assessed the joint effect of the BTEX-H cumulative maximum *mixture* using quantile g-computation (Supplementary Table 4), we found that a one quartile simultaneous increase of each chemical was associated with 10% increase in hypertension incidence (HR: 1.10, 95% CI: 1.07, 1.14).

**Fig. 2:**
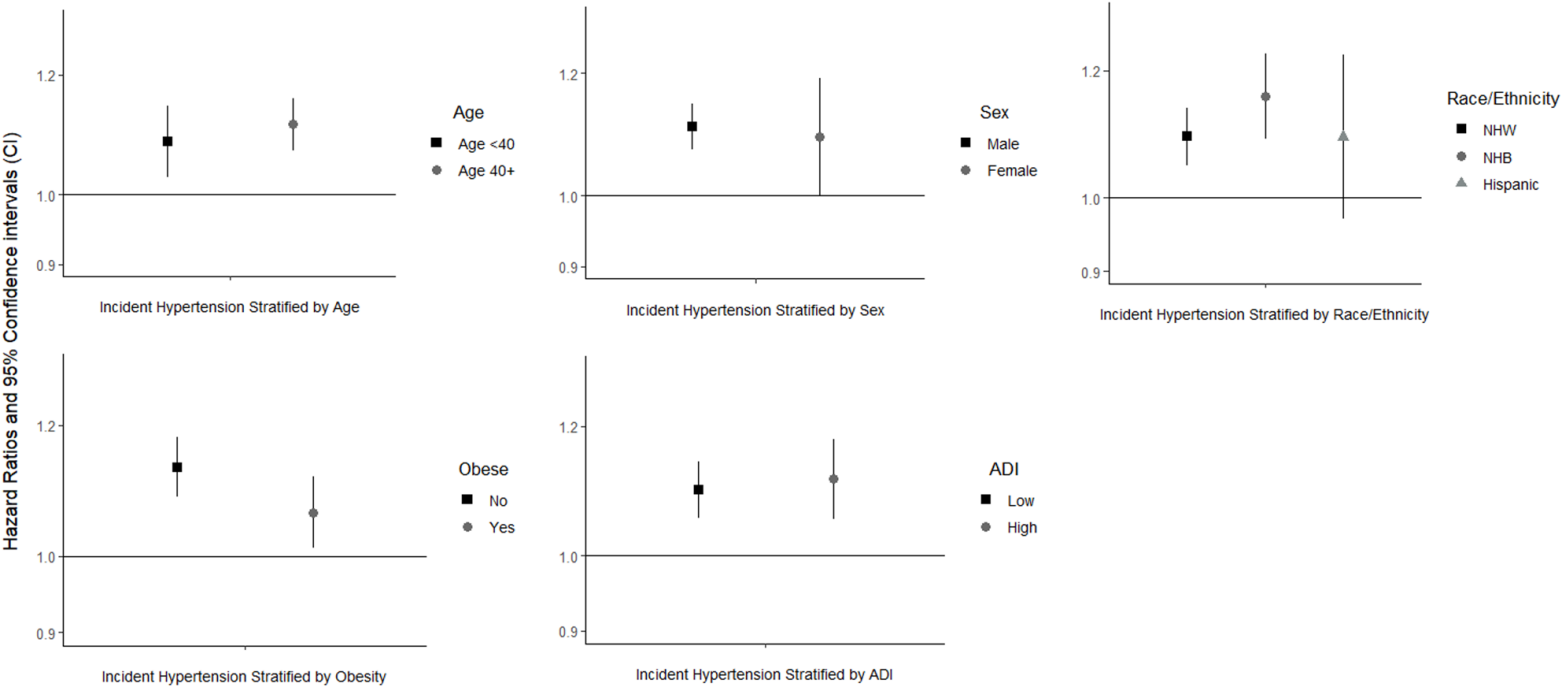
Quantile g-computation estimates for change in hazard of incident hypertension per one quartile increase in *cumulative maximum* exposure to the BTEX-H chemical *mixture* among *DWH* disaster oil spill response and cleanup workers – stratified by age, sex, race and ethnicity, obesity, and Area Deprivation Index.^a, b,c.^ ^a^NHW = Non-Hispanic White; NHB = Non-Hispanic Black ^b^Obesity defined based on body mass index: <30 kg/m^2^ (not obese) and ≥ 30 kg/m^2^ (obese) ^c^Neighborhood disadvantage defined using 2013 Area Deprivation Index (ADI) for low (US percentile 1-75th) and high (US percentile 76-100th) neighborhood disadvantage

We found modestly stronger associations among non-obese (versus obese) individuals for the BTEX-H *mixture* (Fig. 2) and in the top quartiles of most individual BTEX-H chemicals and total BTEX-H (Supplementary Fig. 4). Further, we observed more consistent exposure-response trends among non-obese participants. Stratified analyses did not suggest differences by age, sex, race, or neighborhood disadvantage for hazard of incident hypertension in single agent or mixture models (Fig. 2; Supplementary Figs. 1–3, 5). However, we observed more consistent exposure-response trends among males for benzene, toluene, and ethylbenzene exposures than among females (Supplementary Fig. 2).

Although results were slightly attenuated, interpretations of results did not change when using cumulative average (instead of cumulative maximum) exposures (Supplementary Table 5). Overall, we found no meaningful changes in separate sensitivity analyses for total BTEX-H and the BTEX-H *mixture* in which we excluded individuals who provided discrepant or no information on their hypertension diagnosis date, started the risk period at enrollment, used IPCW, excluded those with any cleanup-related PM_2.5_ exposure from burning/flaring the oil, or restricted analyses to never smokers (Supplementary Tables 6–7). When we applied discrete time assumptions for hypertension diagnosis dates, we observed slightly elevated HRs compared to the primary results, but this did not change overall interpretation of our findings. Similar results were observed for each individual BTEX-H chemical in sensitivity analyses *(data not shown)*.

## Discussion

We examined the association of oil spill cleanup-related individual BTEX-H constituents, total BTEX-H, and the BTEX-H *mixture* with self-reported incident hypertension among OSRC workers up to 11 years after the *DWH* disaster. Individual and total BTEX-H exposures were associated with risk of incident hypertension, with evidence of exposure-response trends for increasing quartiles of exposure. We observed stronger associations among non-obese workers.

Most studies have shown that THC and BTEX-H exposures are positively associated with hypertension cross-sectionally and over a short time period [11–15, 21]. Prior GuLF Study analyses were based on examiner-measured blood pressure among participants up to 3 years after the *DWH* disaster [13, 21]. Two of the non-GuLF Study studies examined ambient BTEX exposure in relation to elevated blood pressure among large samples of pregnant women; however, these results may not be generalizable to the predominantly male GuLF Study cohort or to a wider population with hypertension [11, 12]. Further, BTEX exposures in these two studies were only measured up to 3 months prior to conception [11, 12]. One study examined ambient benzene and, more broadly, diesel emissions (using the 2005 EPA National Air Toxics Assessment database) cross-sectionally among a cohort of women from the Sister Study and found that higher levels of exposure were positively associated with hypertension [15]. Another study, conducted among Black adults who participated in the Jackson Heart Study (JHS), found that urinary metabolites of benzene, toluene, and xylene were associated with slightly higher systolic blood pressure, but not diastolic blood pressure or risk of hypertension [14]. However, the JHS analysis was cross-sectional and utilized urinary VOC metabolites, which reflect only very recent exposures due to the short half-lives of these chemicals [39–41]. Our study builds on existing literature by investigating associations of BTEX-H constituents individually and as a mixture with incident hypertension up to 11 years after exposure. Potential biological mechanisms through which BTEX-H exposure may increase blood pressure include systemic inflammation, endothelial and vascular dysfunction, autonomic nervous system imbalance, genomic instability, and gene expression alterations [42, 43]. One study suggested that benzene exposure may contribute to decreased circulating angiogenic cells, which are predictive of CVD risk [25]. 

Some studies examined VOC exposures more broadly, instead of individual chemical constituents, in relation to blood pressure and hypertension [22–24]. One was an age- and nationality-matched case-control study that observed a higher prevalence of hypertension among 86 workers occupationally exposed to hydrocarbons and organic solvents compared to 86 unexposed office workers [23]. Two cross-sectional studies in Taiwan, one among 200 homemakers and another among 115 office workers, reported that total VOC exposure was associated with elevated systolic and diastolic blood pressure [22, 24]. The present study expanded on findings from previous studies by examining individual, albeit oil spill-related, chemicals, adjustment for important covariates, larger sample size, and longer follow-up time.

We did not find clear evidence of effect measure modification of BTEX-H associations with incident hypertension by age, sex, race, or neighborhood disadvantage. In the earlier GuLF Study analysis of hypertension and blood pressure, slightly stronger associations between higher THC exposure and hypertension were seen among Non-Hispanic Black (versus Non-Hispanic White) and male (versus female) participants [13]. However, the current study did not observe evidence of effect measure modification by race or sex.

We observed slightly stronger HRs among non-obese individuals in the top quartiles of exposure for individual BTEX-H and total BTEX-H and for the overall BTEX-H *mixture*. In contrast, both the prior GuLF Study analysis and the study among office workers in Taiwan observed stronger associations of THC with hypertension and of total VOCs with higher blood pressure, respectively, among overweight and obese individuals [13, 22]. The other GuLF Study analysis of BTEX-H associations with hypertension and measured blood pressure up to 3 years after exposure did not observe evidence of effect measure modification by obesity [21]. Authors of a study among participants from the 1999 and 2000 National Health and Nutrition Examination Survey have suggested that higher levels of BTEX observed among women (versus men) and those with BMI greater than 30 kg/m^2^ (versus BMI < 25 and BMI 25–30 kg/m^2^) may be due to higher levels of body fat in these groups [44], consistent with the lipophilic properties of benzene, toluene, and xylene [39–41]. The conflicting findings from our study may be partially explained by the longer follow-up time. Of note, a GuLF Study analysis of the association of THC and BTEX-H exposures with incident coronary heart disease (CHD) also found stronger associations among non-obese individuals [20]. In that study, Chen et al. (2023) suggested that obese workers experience more variability in CHD risk due to a greater impact of other lifestyle factors such as physical activity and diet, which might obscure an association between spill-related exposures and CHD [20]. This may also explain the stronger HRs seen among non-obese participants in our analysis. The current study lacked information on other lifestyle factors that may influence obesity and hypertension risk such as diet, food access and environment, or genetic factors.

One limitation of our study is the potential for outcome misclassification since hypertension diagnosis relied on self-reports. Prior research has demonstrated that self-reported hypertension has moderate sensitivity (36–92%) but high specificity (94–99%) relative to measured blood pressure [45, 46]. We believe this to be the case in the GuLF Study population, as only 40% of those classified in our earlier study as hypertensive based on measured blood pressure reported use of antihypertensive medications at the time of the study exam [13]. Barriers to health care access (e.g. financial or geographic) may contribute to underreported hypertension. Some studies have reported increased sensitivity of self-report for identifying hypertension among older aged (> 55 years) individuals, which would be consistent with somewhat lower sensitivity in our study, given that the mean age of participants was 40 years [45, 46]. However, we expect outcome misclassification (regarding underreported hypertension) to be non-differential with respect to exposure and to, therefore, bias effect estimates towards the null. Additionally, there may be measurement error in the reported hypertension diagnosis date due to inaccurate recall (as illustrated by discrepancies across visits). However, it is reassuring that no notable changes were observed between main results and sensitivity analyses in which we excluded those who provided conflicting hypertension diagnosis dates or no diagnosis date or used discrete time assumptions for hypertension diagnosis dates. We were unable to identify individuals who developed hypertension after beginning oil spill response and cleanup work but did not enroll into the GuLF Study. This left truncation could result in underestimation of the true HRs since hypertension is a relatively common outcome that develops gradually over time. We started the risk period at enrollment in sensitivity analyses to examine potential bias from this limitation and did not observe meaningful changes in effect estimates. Finally, we did not observe any meaningful changes in effect estimates when we used inverse probability censor weights to account for potential bias from loss to follow-up.

The current study lacked data on other environmental exposures experienced during oil spill response and cleanup work or follow-up. However, in sensitivity analyses, we did not observe meaningful changes in results when we excluded individuals with any cleanup-related PM_2.5_ exposure from controlled burning/flaring. While the exposure estimates likely contain some measurement error, we expect this to be non-differential and to not substantially bias effect estimates since our analysis used exposure quartiles. Additionally, because it was not possible to determine how much time workers who performed multiple jobs or tasks per day spent in each of those jobs/tasks, exposure estimates are not strictly time weighted. Although cumulative daily maximum (main results) would not be affected by the amount of time spent on a task, we examined cumulative daily average exposures in sensitivity analyses. While effect estimates for cumulative average exposures were slightly attenuated compared to cumulative maximum exposures, we did not find any meaningful change in the interpretation of results. Finally, health status may have influenced the cleanup jobs and activities assigned to workers such that healthier workers potentially experienced higher exposures (healthy worker effect). This could potentially contribute to underestimated associations observed in the current study.

A major strength of this study was the use of quantitative BTEX-H exposure estimates based on air measurement data from personal exposure samples obtained in real-time during cleanup from oil spill response and cleanup workers and linked with detailed *DWH* spill work histories provided by GuLF Study participants. The large size of the GuLF Study cohort and the detailed data collected from participants in structured interviews allowed us to account for a range of potential confounders and effect measure modifiers. Additionally, most existing studies have examined hypertension related to short-term effects of BTEX-H exposures, but our study was unique in its ability to investigate this relationship several years after exposure.

In this large longitudinal study, we found that exposures to oil spill cleanup-related individual BTEX-H chemicals, total BTEX-H, and the BTEX-H *mixture* were associated with an elevated risk of hypertension, with evidence of exposure-response trends and stronger associations observed among non-obese workers. Findings from our study build upon prior studies that have shown positive associations of VOC and BTEX-H exposures with hypertension. Exposure to BTEX-H chemicals is widespread in both the occupational and general population setting. Thus, findings from our study may have broader population health implications. Additional studies incorporating a range of exposure levels in different settings and blood pressure monitoring over time are warranted.

## Supplementary Information

Supplementary Material 1.

## Data Availability

The data are not available for on-line replication, but requests for study data to be shared under individualized Data Sharing Agreements may be made through the GuLF Study management site (see instructions at https://gulfstudy.nih.gov/en/forresearchers.html.

## Abbreviations

BTEX-H: Benzene, toluene, ethylbenzene, xylene, n-hexane
ADI: Area Deprivation Index
BMI: Body mass index
BP: Blood pressure
CHD: Coronary heart disease
CI: Confidence interval
CVD: Cardiovascular disease
DWH: Deepwater Horizon
EPA: Environmental Protection Agency
GuLF: Gulf Long-term Follow-up
HR: Hazard ratio
JHS: Jackson Heart Study
LOD: limit of detection
OSRC: oil spill response and cleanup
PM: Particulate matter
ppb: Parts per billion
SD: Standard deviation
THC: Total hydrocarbons
US: United States
VOC: Volatile organic compound
