# Supplementary material for: Association of oil spill cleanup-related hydrocarbon exposure with incident hypertension up to 11 years after exposure in the Gulf Long-term Follow-up Study
Source: Environ Health. 2025 Dec 30;25:7. doi: 10.1186/s12940-025-01253-9 (PMC12860164; doi:10.1186/s12940-025-01253-9)
Supplement: Supplementary file 1 — Supplementary Material 1. [file 12940_2025_1253_MOESM1_ESM.docx]

**Association of oil spill cleanup-related hydrocarbon exposure with incident hypertension up to 11 years after exposure in the Gulf Long-term Follow-up Study**

Opal P. Patel, Jessie K. Edwards, Anna M. Kucharska-Newton, Eric A. Whitsel, Kate Christenbury, W. Braxton Jackson II, Kaitlyn G. Lawrence, Patricia A. Stewart, Mark R. Stenzel, Lawrence S. Engel*, Dale P. Sandler* (*co-senior authors)

**Supplementary Material**

Supplementary Table 1: Distribution of cumulative daily maximum exposure among GuLF Study participants (N=18,340)

| *Parts per billion* | Minimum | Q2 cut point | Q3 cut point | Q4 cut point | Maximum |
| --- | --- | --- | --- | --- | --- |
| Benzene | 0.01 | 64.90 | 314.58 | 960.88 | 9,863.37 |
| Toluene | 0.12 | 238.14 | 1,266.39 | 3,649.35 | 26,724.55 |
| Ethylbenzene | 0.01 | 56.47 | 249.49 | 749.93 | 8,129.90 |
| Xylene | 1.58 | 673.88 | 1,712.19 | 4,140.37 | 24,935.65 |
| n-Hexane | 0.06 | 111.51 | 527.48 | 2,506.56 | 90,157.50 |

Supplementary Table 2: Characteristics of GuLF Study participants at enrollment and the first and second follow-up interviews

|  | **GuLF Study Visit*^a^*** | | |
| --- | --- | --- | --- |
| **Characteristic** | **Enrollment** | **First Follow-up** | **Second Follow-up** |
|  | *n=18,619* | *n=12,604*  *(67.7%)* | *n=8,512*  *(45.7%)* |
|  | ***Mean (SD)*** | ***Mean (SD)*** | ***Mean (SD)*** |
| Age at enrollment (continuous) | 40.0 (12.1) | 41.3 (12.2) | 42.0 (12.2) |
|  | ***n (%)*** | ***n (%)*** | ***n (%)*** |
| Age at enrollment |  |  |  |
| <40 years | 9,572 (51.4) | 5,860 (46.5) | 3,751 (44.1) |
| ≥40 years | 9,047 (48.6) | 6,744 (53.5) | 4,761 (55.9) |
| Sex |  |  |  |
| Male | 15,211 (81.7) | 10,198 (80.9) | 6,768 (79.5) |
| Female | 3,408 (18.3) | 2,406 (19.1) | 1,744 (20.5) |
| Education |  |  |  |
| Less than high school | 2,786 (15.0) | 1,726 (13.7) | 908 (10.7) |
| High school diploma/GED | 5,484 (29.5) | 3,490 (27.7) | 2,115 (24.8) |
| Some college/2 year degree | 5,593 (30.0) | 3,772 (29.9) | 2,581 (30.3) |
| 4 year college graduate or more | 4,756 (25.5) | 3,616 (28.7) | 2,908 (34.2) |
| Race and ethnicity |  |  |  |
| Non-Hispanic White | 12,013 (64.5) | 8,273 (65.6) | 5,994 (70.4) |
| Non-Hispanic Black | 4,149 (22.3) | 2,694 (21.4) | 1,505 (17.7) |
| Non-Hispanic Other | 1,138 (6.1) | 799 (6.3) | 500 (5.9) |
| Hispanic White | 417 (2.2) | 284 (2.3) | 190 (2.2) |
| Hispanic Black | 72 (0.4) | 47 (0.4) | 27 (0.3) |
| Hispanic Other | 830 (4.5) | 507 (4.0) | 296 (3.5) |
| Smoking status |  |  |  |
| Never smoker | 9,120 (49.6) | 6,365 (51.0) | 4,525 (53.6) |
| Former smoker | 3,626 (19.7) | 2,539 (20.4) | 1,830 (21.7) |
| Light current smoker | 3,807 (20.7) | 2,396 (19.2) | 1,418 (16.8) |
| Heavy current smoker | 1,837 (10.0) | 1,172 (9.4) | 665 (7.9) |
| BMI (kg/m^2^)*^b^* |  |  |  |
| <18.5 | 146 (0.8) | 101 (0.8) | 62 (0.7) |
| 18.5-24.9 | 5,510 (29.6) | 3,648 (28.9) | 2,438 (28.6) |
| 25-29.9 | 7,823 (42.0) | 5,312 (42.1) | 3,667 (43.1) |
| ≥30 | 5,140 (27.6) | 3,543 (28.1) | 2,345 (27.5) |
| Neighborhood Disadvantage*^c^* |  |  |  |
| Low | 13,747 (73.8) | 9,372 (74.4) | 6,574 (77.2) |
| High | 4,872 (27.2) | 3,232 (26.5) | 1,938 (23.5) |
| Risk period*^d^* |  |  |  |
| 0-1.9 years | 6,333 (34.0) | 1,770 (14.0) | 1,049 (12.3) |
| 2-3.9 years | 4,123 (22.1) | 3,545 (28.1) | 509 (6.0) |
| 4-5.9 years | 1,444 (7.8) | 1,338 (10.6) | 494 (5.8) |
| ≥6 years | 6,719 (36.1) | 5,951 (47.2) | 6,460 (75.9) |
| *^a^*Enrollment interview occurred from March 2011 to March 2013. First follow-up interview occurred from May 2013 to April 2016. Second follow-up interview occurred from November 2017 to July 2021.  *^b^*BMI = body mass index; calculated as weight in kilograms divided by height in meters squared (kg/m^2^) as reported at enrollment.  *^c^*Neighborhood disadvantage defined using the 2013 Area Deprivation Index (ADI) for low (US percentile 1-75^th^) and high (US percentile 76-100^th^) neighborhood disadvantage.  *^d^*The risk period (years) was the time between each participant’s end of cleanup date to the first self-reported hypertension diagnosis date, death from other causes, and end of cohort follow-up. | | | |

Supplementary Table 3: Spearman correlation coefficients between BTEX-H oil spill chemicals among GuLF Study participants (N=18,340)

| ***Cumulative maximum*** | Benzene | Toluene | Ethylbenzene | Xylene | n-Hexane |
| --- | --- | --- | --- | --- | --- |
| Benzene | 1.0 |  |  |  |  |
| Toluene | 0.95 | 1.0 |  |  |  |
| Ethylbenzene | 0.94 | 0.93 | 1.0 |  |  |
| Xylene | 0.90 | 0.91 | 0.95 | 1.0 |  |
| n-Hexane | 0.88 | 0.89 | 0.91 | 0.87 | 1.0 |

Supplementary Table 4: Associations between *cumulative maximum* exposure to BTEX-H chemicals and hazard of hypertension among *DWH* disaster oil spill response and cleanup workers (N=18,619)

| **Exposure*^a^*** | **Q1*^b^*** | **Q2** |  | **Q3** |  | **Q4** |  | **p-trend** |
| --- | --- | --- | --- | --- | --- | --- | --- | --- |
|  |  | ***HR*** | ***95% CI*** | ***HR*** | ***95% CI*** | ***HR*** | ***95% CI*** |  |
| **total BTEX-H** | Ref | 1.09 | (1.00, 1.20) | 1.17 | (1.07, 1.29) | 1.31 | (1.19, 1.44) | <0.01 |
| **Benzene** | Ref | 1.15 | (1.05, 1.26) | 1.22 | (1.11, 1.34) | 1.30 | (1.18, 1.43) | <0.01 |
| **Toluene** | Ref | 1.17 | (1.07, 1.28) | 1.16 | (1.06, 1.27) | 1.35 | (1.23, 1.49) | <0.01 |
| **Ethylbenzene** | Ref | 1.08 | (0.99, 1.18) | 1.20 | (1.09, 1.31) | 1.30 | (1.18, 1.42) | <0.01 |
| **Xylene** | Ref | 1.02 | (0.93, 1.11) | 1.14 | (1.04, 1.25) | 1.27 | (1.16, 1.39) | <0.01 |
| **n-Hexane** | Ref | 1.10 | (1.01, 1.21) | 1.15 | (1.05, 1.26) | 1.33 | (1.21, 1.45) | <0.01 |
|  |  | ***HR*** | ***95% CI*** |  |  |  |  |  |
| **BTEX-H *mixture^c^*** |  | 1.10 | (1.07, 1.14) |  |  |  |  |  |
| *^a^*Cumulative exposure to each BTEX-H chemical measured in ppb-days, calculated as the sum of the daily maximum exposure estimates. Total BTEX-H calculated as a sum of the individual BTEX-H ppb values.  *^b^*Multivariable Cox proportional hazards regression models adjusted for age (with quadratic splines at ages 50 and 60 years), sex, self-reported race, Hispanic ethnicity, highest educational attainment, BMI, and residential proximity to the oil spill.  *^c^*Quantile g-computation estimates for hazard of incident hypertension per one quartile increase in *cumulative maximum* exposure to all crude oil chemicals (BTEX-H). | | | | | | | | |

Supplementary Table 5: Associations between *cumulative average* exposure to BTEX-H chemicals and hazard of hypertension among *DWH* disaster oil spill response and cleanup workers (N=18,619)

| **Exposure*^a^*** | **Q1*^b^*** | **Q2** |  | **Q3** |  | **Q4** |  | **p-trend** |
| --- | --- | --- | --- | --- | --- | --- | --- | --- |
|  |  | ***HR*** | ***95% CI*** | ***HR*** | ***95% CI*** | ***HR*** | ***95% CI*** |  |
| **total BTEX-H** | Ref | 1.06 | (0.97, 1.16) | 1.12 | (1.03, 1.23) | 1.20 | (1.10, 1.32) | <0.01 |
| **Benzene** | Ref | 1.17 | (1.07, 1.28) | 1.22 | (1.12, 1.34) | 1.19 | (1.08, 1.30) | <0.01 |
| **Toluene** | Ref | 1.11 | (1.01, 1.21) | 1.17 | (1.07, 1.29) | 1.18 | (1.07, 1.30) | <0.01 |
| **Ethylbenzene** | Ref | 1.11 | (1.01, 1.22) | 1.14 | (1.04, 1.25) | 1.23 | (1.12, 1.35) | <0.01 |
| **Xylene** | Ref | 1.07 | (0.98, 1.16) | 1.06 | (0.97, 1.16) | 1.19 | (1.09, 1.31) | <0.01 |
| **n-Hexane** | Ref | 1.10 | (1.00, 1.20) | 1.13 | (1.03, 1.24) | 1.23 | (1.12, 1.34) | <0.01 |
|  |  | ***HR*** | ***95% CI*** |  |  |  |  |  |
| **BTEX-H *mixture^c^*** |  | 1.07 | (1.04, 1.10) |  |  |  |  |  |
| *^a^*Cumulative exposure to each BTEX-H chemical measured in ppb-days, calculated as the sum of the daily average exposure estimates. Total BTEX-H calculated as a sum of the individual BTEX-H ppb values.  *^b^*Multivariable Cox proportional hazards regression models adjusted for age (with quadratic splines at ages 50 and 60 years), sex, self-reported race, Hispanic ethnicity, highest educational attainment, BMI, and residential proximity to the oil spill.  *^c^*Quantile g-computation estimates for hazard of incident hypertension per one quartile increase in *cumulative average* exposure to all crude oil chemicals (BTEX-H). | | | | | | | | |

Supplementary Table 6: Results of associations between *cumulative maximum* total BTEX-H exposure and incident hypertension in main versus sensitivity analyses

| **Model*^a^*** | **Q1** | **Q2** | **95% CI** | **Q3** | **95% CI** | **Q4** | **95% CI** |
| --- | --- | --- | --- | --- | --- | --- | --- |
| Main adjusted model*^b^* | Ref | 1.09 | (1.00,1.20) | 1.17 | (1.07,1.29) | 1.31 | (1.19,1.44) |
| Excluded discrepant/no hypertension diagnosis date | Ref | 1.02 | (0.91,1.14) | 1.14 | (1.02,1.28) | 1.25 | (1.12,1.41) |
| Discrete time assumption for hypertension diagnosis date | Ref | 1.14 | (1.03,1.27) | 1.24 | (1.12,1.37) | 1.47 | (1.32,1.63) |
| Started risk period at enrollment | Ref | 1.14 | (1.01,1.28) | 1.26 | (1.11,1.42) | 1.33 | (1.17,1.51) |
| Excluded workers with any cleanup-related PM_2.5_ exposure | Ref | 1.10 | (1.00,1.20) | 1.14 | (1.04,1.25) | 1.29 | (1.17,1.43) |
| Restricted to never smokers at enrollment | Ref | 1.18 | (1.03,1.35) | 1.17 | (1.02,1.34) | 1.39 | (1.21,1.60) |
| Inverse probability of censoring weighting | Ref | 1.09 | (0.99, 1.19) | 1.18 | (1.07, 1.30) | 1.31 | (1.18, 1.44) |
| ^a^Cumulative exposure to each BTEX-H chemical measured in ppb-days, calculated as the sum of the daily maximum exposure estimates. Total BTEX-H calculated as a sum of the individual BTEX-H ppb values.  ^b^Main Cox proportional hazards regression models adjusted for age (with linear spline at ages 50 and 60 years), sex, self-reported race, Hispanic ethnicity, highest educational attainment, diabetes prior to the spill, and residential proximity to the oil spill. | | | | | | | |

Supplementary Table 7: Results of quantile g-computation estimates for hazard of incident hypertension per one quartile increase in *cumulative maximum* exposure to the BTEX-H chemical ­*mixture* in main versus sensitivity analyses

|  | **BTEX-H *Mixture*** | |
| --- | --- | --- |
| **Model*^a^*** | ***HR*** | ***95% CI*** |
| Main adjusted model*^b^* | 1.10 | (1.07,1.14) |
| Excluded discrepant/no hypertension diagnosis date | 1.10 | (1.05,1.14) |
| Discrete time assumption for hypertension diagnosis date | 1.14 | (1.10,1.19) |
| Started risk period at enrollment | 1.12 | (1.07,1.17) |
| Excluded workers with any cleanup-related PM_2.5_ exposure | 1.09 | (1.06,1.13) |
| Restricted to never smokers at enrollment | 1.12 | (1.07,1.18) |
| *^a^*Cumulative exposure to each BTEX-H chemical measured in ppb-days, calculated as the sum of the daily maximum exposure estimates.  *^b^*Main Cox proportional hazards regression models adjusted for age (with linear spline at ages 50 and 60 years), sex, self-reported race, Hispanic ethnicity, highest educational attainment, BMI, and residential proximity to the oil spill. | | |


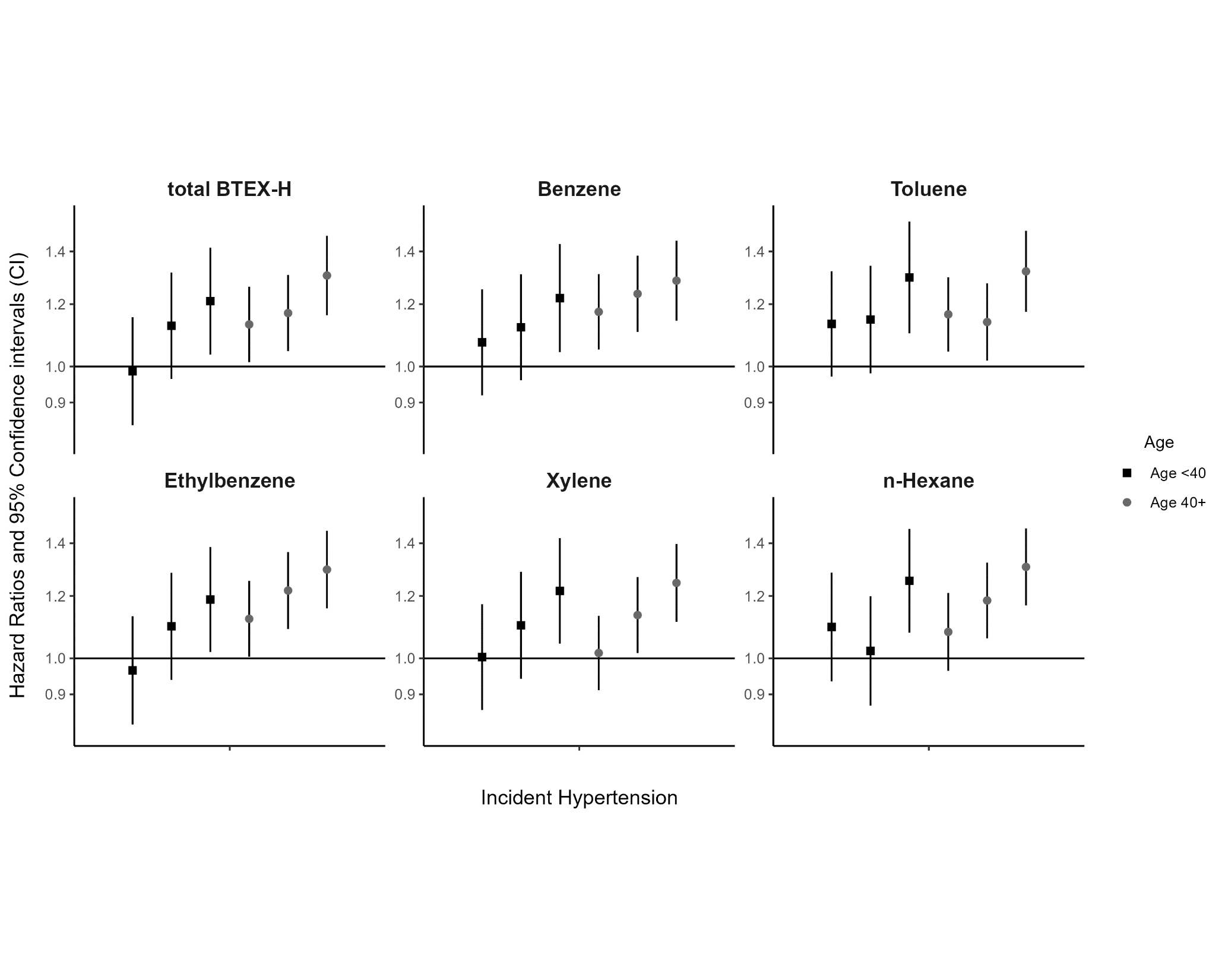


Supplementary Figure 1: Associations between *cumulative maximum* exposure to BTEX-H chemicals and hazard of hypertension among *DWH* disaster oil spill response and cleanup workers – stratified by age (<40 vs. ≥40 years)


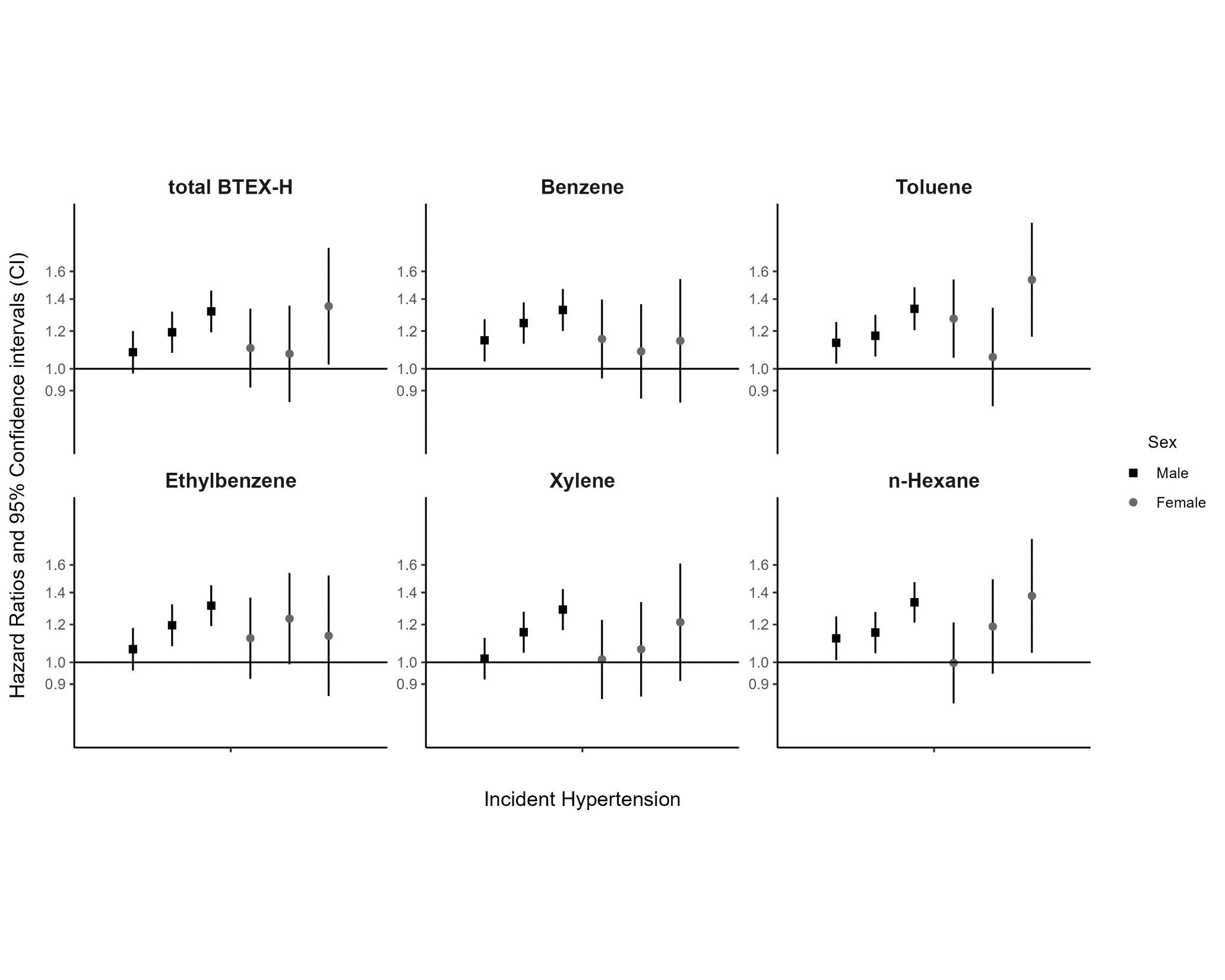


Supplementary Figure 2: Associations between *cumulative maximum* exposure to BTEX-H chemicals and hazard of hypertension among *DWH* disaster oil spill response and cleanup workers – stratified by sex (male vs. female)


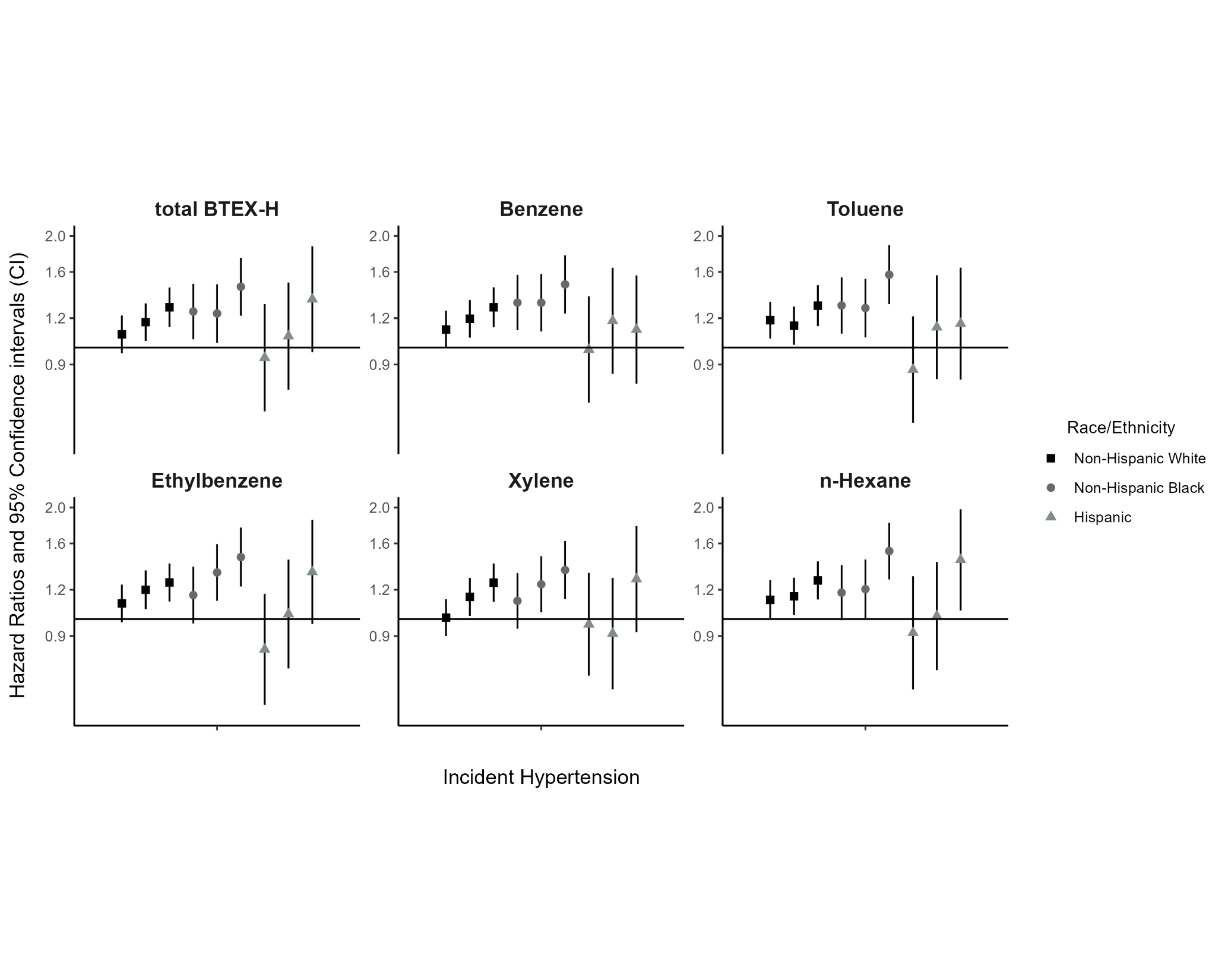


Supplementary Figure 3: Associations between *cumulative maximum* exposure to BTEX-H chemicals and hazard of hypertension among *DWH* disaster oil spill response and cleanup workers – stratified by race (Non-Hispanic White, Non-Hispanic Black, and Hispanic)


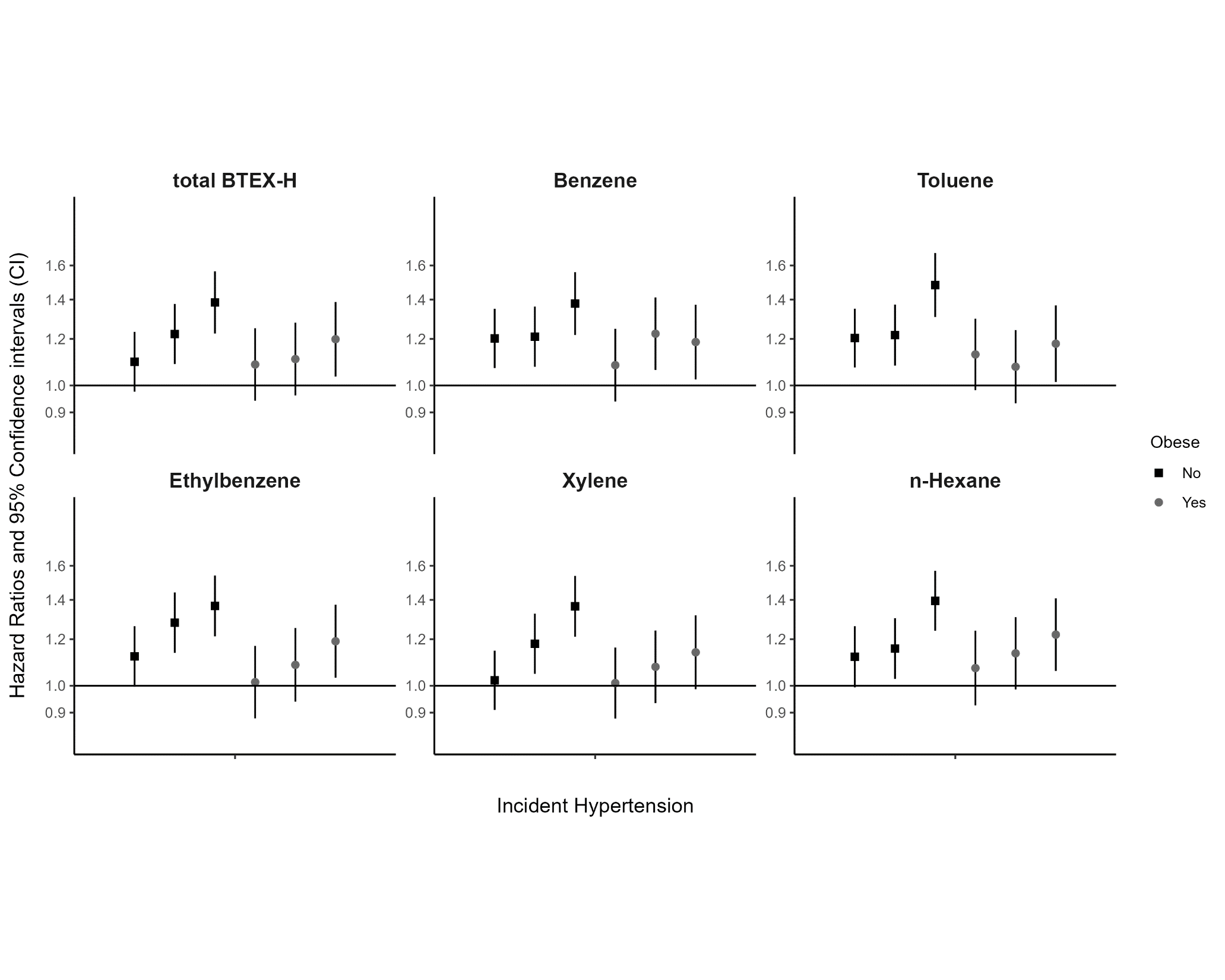


Supplementary Figure 4: Associations between *cumulative maximum* exposure to BTEX-H chemicals and hazard of hypertension among *DWH* disaster oil spill response and cleanup workers – stratified by obesity (non-obese vs. obese)


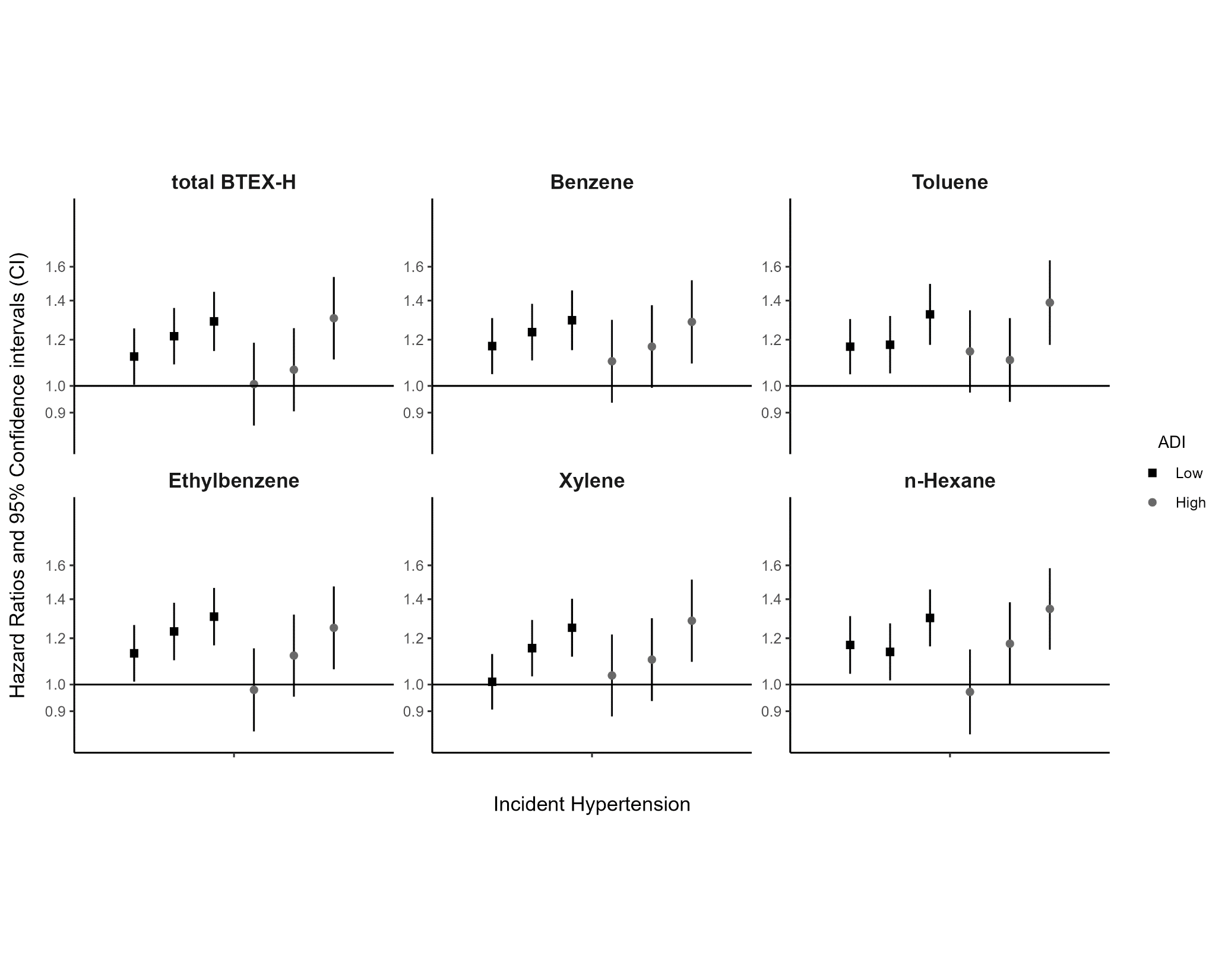


Supplementary Figure 5: Associations between *cumulative maximum* exposure to BTEX-H chemicals and hazard of hypertension among *DWH* disaster oil spill response and cleanup workers – stratified by Area Deprivation Index (low vs. high neighborhood disadvantage)
